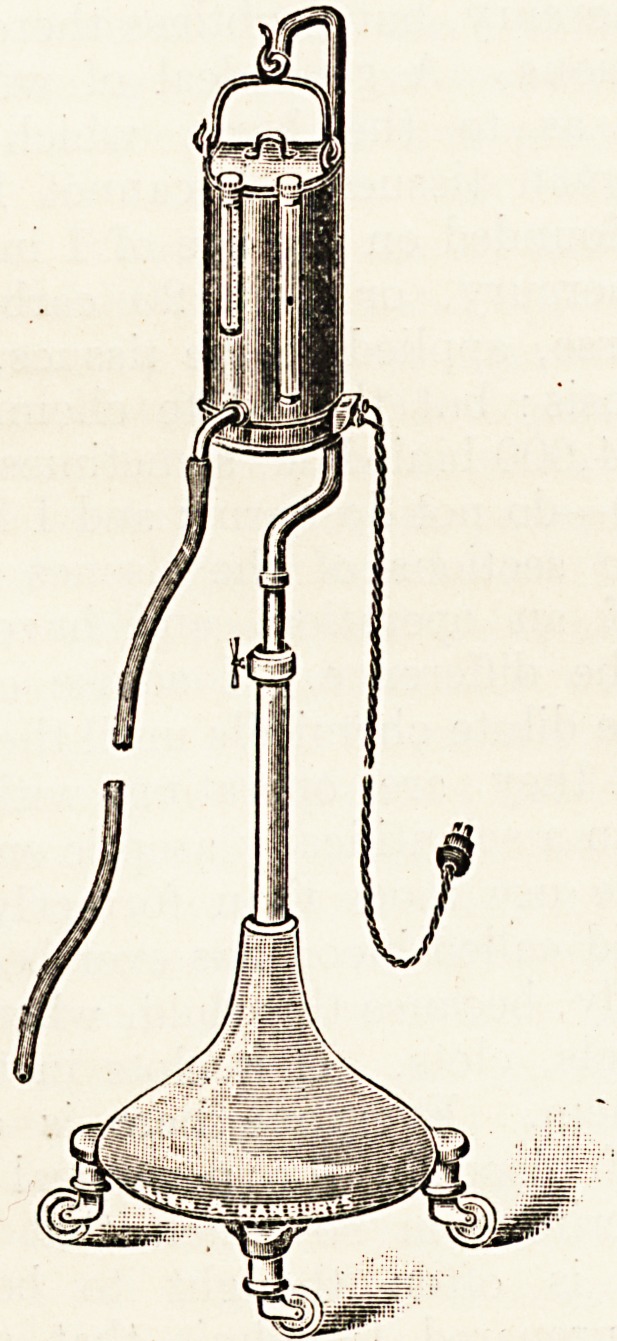# Note on Continuous Proctoclysis

**Published:** 1909-10-16

**Authors:** Herbert J. Paterson


### NOTE ON CONTINUOUS PROCTOCLYSIS.

By HERBERT J. PATERSON, M.B., B.C., Cantab., F.R.C.S.

Not the least important of the many good things which have come from America is the plan of continuous administration by the rectum of warm saline solution, as introduced by Dr. J. B. Murphy. This method of treatment is, in my opinion, one of the greatest advances in abdominal surgery which has been made within recent years. Its routine adoption leads, I believe, to more rapid, and certainly to more comfortable, convalescence after coeliotomy, and its beneficial effects in cases of general septic peritonitis would have surprised me, had I not been prepared for them by hearing from Dr. Murphy's own lips of the wonderful results which he has obtained in the treatment of this serious condition, since the adoption of the practice of continuous proctoclysis.

Success in using the Murphy method depends on attention to detail, and the two most important points are: first, the regulation of the flow from the supply-can by gravity alone, and not by constriction of the delivery tube; and, secondly, the maintenance of the saline at a constant and appropriate temperature. To obviate the latter difficulty I have had an apparatus made which does away with the necessity for constant supervision. The apparatus consists of an electroplated douche can which holds about five pints. In the front of the can are a thermometer and a gauge glass, by the side of which the can is graduated in half-pints, so that the amount of saline entering the rectum can be readily estimated. The saline leaves the can through a delivery tube with a half-inch bore, to which is attached three feet of rubber tubing connected with a large rectal tube. Under the bottom of the can is an electric heater, which can be connected with any electric supply of suitable voltage by means of a flexible cord and wall plug. The can is suspended on an adjustable stand mounted on castors so that it can be readily wheeled up to the bedside. I have found by experiment that with a ward temperature of from 65° to 70° F. the solution in the can must be kept at a temperature of 106° F. in order to ensure that the saline enters the rectum at a temperature of from 99° to 100° F. The electric heater is so adjusted that if the saline solution is put into the can at a temperature of 106° F. the temperature remains almost constant so long as the current is switched on. The apparatus which has been made for me by Messrs. Allen and Hanburys answers its purpose admirably, and may be left for several hours without any attention.

## Figures and Tables

**Figure f1:**